# Effects of Chitosan Oligosaccharide Supplementation on Lipid Profiles in Overweight and Obese Thai Women: A Randomized Controlled Trial

**DOI:** 10.1002/fsn3.71369

**Published:** 2025-12-18

**Authors:** Natnaree Mayang, Kitti Sranacharoenpong, Siriyupa Netramai, Korapat Mayurasakorn, Magnus Bergkvist

**Affiliations:** ^1^ School of Bioinnovation and Bio‐Based Product Intelligence, Faculty of Science Mahidol University Bangkok Thailand; ^2^ School of Bioinnovation and Bio‐Based Product Intelligence, Faculty of Science Mahidol University Nakhon Pathom Thailand; ^3^ ASEAN Institute for Health Development Mahidol University Nakhon Pathom Thailand; ^4^ Siriraj Population Health and Nutrition Research Group (SPHERE), Research Group and Research Network Division, Research Department, Faculty of Medicine Siriraj Hospital Mahidol University Bangkok Thailand; ^5^ Global Innovation Center Thai Union Group PCL Bangkok Thailand

**Keywords:** chitosan oligosaccharides, lipid profile, obesity, overweight

## Abstract

Obesity represents a significant public health challenge closely associated with dyslipidaemia. The objective of this study was to evaluate the impact of chitosan oligosaccharides (COS) supplementation on lipid profiles and body composition among overweight or obese Thai women. A randomized, double‐blind, placebo‐controlled trial was conducted over a duration of 60 days, involving 60 participants. The participants were assigned to one of three groups: one group received 1.5 g of COS daily (1.5COS, *n* = 20), another group received 3 g of COS daily (3COS, *n* = 20), and a control group received a placebo (*n* = 20). Biochemical and anthropometric data were collected prior to and following the intervention, while dietary intake was monitored throughout the study. The intention‐to‐treat analysis indicated that no statistically significant differences in lipid profiles were noted between the COS and placebo groups. However, the 1.5COS group exhibited a significant increase in mean high‐density lipoprotein (HDL) levels (*p* = 0.024). Additionally, the waist‐to‐hip ratio reduction was observed as a within‐group change in the 3 COS group, not a statistically significant between‐group difference. The findings of this trial did not indicate a clinically significant reduction in lipid profiles attributable to COS supplementation.

**Trial Registration:** Thai Clinical Trials Registry: TCTR20250625005 on 25 June 2025

## Graphical abstract

This randomized, double‐blind, placebo‐controlled clinical trial investigates the effects of low‐molecular‐weight chitosan oligosaccharides (COS) supplementation on lipid profiles and body composition in overweight and obese Thai women over a 60‐day intervention period. Our findings suggest that COS at a daily dosage of 1.5 g may significantly improve certain lipid parameters (notably HDL level) without adverse effects.

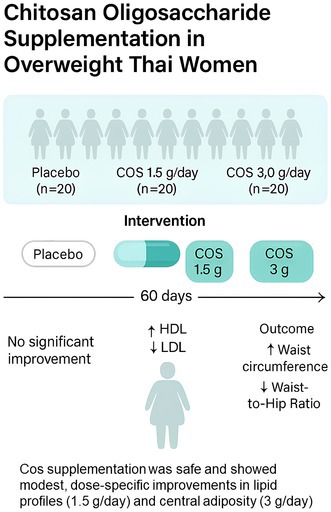

## 1 Introduction

The prevalence of overweight and obesity has continued to rise globally (Luhar et al. 2020; Sakboonyarat et al. 2020; World Health Organization 2024). According to data from the World Health Organization (WHO), both adult and child populations are experiencing rising rates of these conditions. Specifically, in 2016, more than 650 million adults, which equates to approximately 13.0% of the global adult population (11.0% of men and 15.0% of women), were classified as obese (World Health Organization 2024). In Thailand, the incidence of obesity saw an increase from 34.0% in 2012 to 45.0% in 2018. By 2022, approximately 2.5 billion adults were categorized as overweight, with 890 million individuals living with obesity (World Health Organization 2024). Notably, the prevalence of obesity among women escalated from 42.0% to 51.0%, while among men, it rose from 24.0% to 33.0% from 2012 to 2018 (Sakboonyarat et al. 2020).

Chitosan oligosaccharide, commonly referred to as chito‐oligosaccharides (COS), is derived from chitin or high molecular weight chitosan, which is predominantly sourced from the shells of crustaceans and insects (Naveed et al. 2019). Research focusing on the effects of COS on serum lipid reduction in humans remains limited. To date, only one study has investigated the potential impact of COS with a molecular weight (Mw) of less than 1 kDa on serum lipid levels in humans (Choi et al. 2012). Nonetheless, several animal studies have indicated that COS may facilitate weight reduction and lower serum lipid levels, exhibiting effects comparable to those of Orlistat (Huang et al. 2015; Wang et al. 2019). In contrast, chitosan, a related compound, has undergone more comprehensive examination regarding its potential effects. A study involving human subjects conducted by Chirdkiatisak et al. (2019) suggested that chitosan may contribute to reductions in body weight and serum lipids. However, contradictory findings from other research indicate that chitosan may not significantly affect these clinical outcomes (Bokura and Kobayashi 2003; Jaffer and Sampalis 2007). Consequently, the present study aims to investigate the effects of COS supplementation on serum lipid profiles and body composition in overweight or obese Thai women.

## 2 Methods

This study was conducted at the Faculty of Medicine Siriraj Hospital, located in Bangkok, Thailand. The study protocol and associated ethical documentation obtained approval (MU‐MOU 2021/457.2610).

### 2.1 Study Design

The design of this study was a 60‐day, double‐blind, placebo‐controlled randomized trial. Participants were randomly allocated into three groups, with 22 individuals in each group. The groups were assigned to receive COS at dosages of 3 g/day, 1.5 g/day, or a placebo. Participants ingested two supplement capsules per meal, three times daily, approximately 10–15 min prior to each meal. Eligibility for participation in the double‐blind, 60‐day randomized intervention phase was restricted to individuals meeting the specified criteria for lipid profiles along with a classification of overweight or obesity.

### 2.2 Study Participants

**FIGURE 1:**
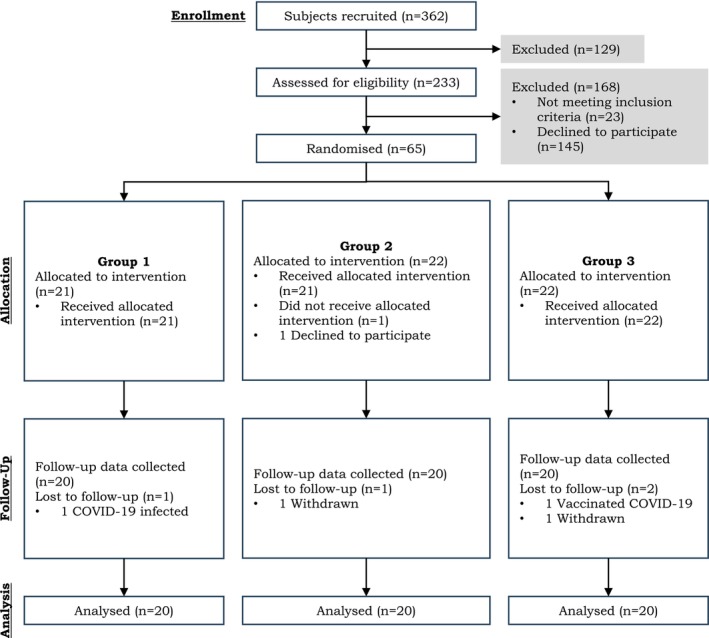
Diagram of subjects enrolled in the study.

Study participants were recruited through poster advertisements disseminated in hospitals and various university campuses (Figure 1). All female participants provided written informed consent and underwent verbal screening conducted online. The inclusion criteria were as follows: (1) Thai women with a BMI ≥ 23.0 kg/m^2^; (2) waist circumference ≥ 80.0 cm; (3) age ranging from 30 to 59 years; (4) total cholesterol level exceeding 5.2 mmol/L or LDL‐C level exceeding 3.4 mmol/L; and (5) capacity to take food photographs and utilize the LINE application for information submission. Participants were required to provide blood samples for lipid profile analyses, with results needing to meet specific criteria. The exclusion criteria included: (1) a history of metabolic syndrome, or any hepatic, renal, autoimmune, gastrointestinal, or neurological diseases; (2) current or recent use of food supplements, over‐the‐counter products, and/or pharmaceutical agents that may affect the study outcomes (e.g., Orlistat); (3) current or recent instances of eating disorders, bipolar disorder, or severe depression; (4) pregnancy or lactation; (5) allergy or intolerance to crustaceans and/or seafood products; (6) vegetarian dietary preferences; (7) current COVID‐19 patients; (8) recovery from COVID‐19 within the previous 3 months; and (9) individuals deemed unlikely to adhere to study treatment and follow‐up protocols.

### 2.3 Randomization, Dosing, and Dispensing

Participants who satisfied the inclusion criteria during screening were then scheduled for clinic visits in accordance with the study protocol. The initial visit took place following the screening process, adhering to established inclusion criteria. A total of 88 participants were enrolled for the collection of blood samples and measurements of body weight, height, waist circumference, and blood pressure. Blood samples were procured following a 12‐h overnight fasting period. Serum lipid profiles, including total cholesterol, HDL cholesterol, and triacylglycerol, were analyzed utilizing enzymatic colorimetric tests, while LDL‐C levels were calculated using the Friedwald equation. Plasma glucose levels were determined through enzymatic colorimetric assays. Additionally, liver function tests were administered to evaluate liver enzymes, such as aspartate aminotransferases (AST) and alanine aminotransferase (ALT). Body weight was measured employing calibrated digital scales (Seca, Model 708, Germany), accurate to the nearest 0.1 kg, while height measurements were conducted by an expert using a wall‐mounted stadiometer (Seca, Model 222, Germany). Waist circumference was recorded to the nearest 0.1 cm, measured midway between the last rib and the crest of the ileum at the natural narrowing of the waist, utilizing a non‐stretch tape measure on lightly clad participants. Blood pressure was assessed on the non‐dominant arm after a 5‐min rest period.

Ultimately, a total of 65 participants fulfilled the eligibility criteria for inclusion in this study, as determined through biochemical evaluations and the defined inclusion criteria. All participants were randomly assigned in a 1:1:1 ratio to receive one of three interventions: 1.5 g of COS per day, 3 g of COS per day, or a placebo. The supplement utilized in the study was COS with a molecular weight of less than 1 kDa, sourced from Fair Medicine Co. Ltd., and subsequently formulated into capsule pills by the same organization. Additionally, the placebo capsules, each containing 500 mg of rice flour, were prepared by the same company.

Participants received instructions to take two capsules prior to each of their three main daily meals, for a total of six capsules per day, irrespective of whether they were assigned to COS or the placebo. The study center dispensed the supplements under blinded conditions. The randomization sequence was generated based on cholesterol levels and age groups. Throughout the study, the treatment group assignments remained undisclosed to both the researchers and data entry personnel, with central management conducted by the statistician.

### 2.4 Interventions

A total of 65 participants satisfied the eligibility criteria for inclusion in this study. These criteria were established based on biochemical assessments alongside predefined inclusion parameters. The study was designed as a 60‐day, double‐blinded, placebo‐randomized controlled trial. Participants were allocated to three groups using block randomization and masking techniques, with each group receiving either a daily dosage of 1.5 g of COS, 3 g of COS, or a placebo. Throughout the 60‐day period, participants were required to ingest six capsules each day, irrespective of whether they were assigned to the COS or placebo groups, prior to each of their three daily meals.

At baseline, data collection encompassed general demographic information, nutritional status, 24‐h dietary recall, a physical activity questionnaire (Pan et al. 2016), blood biochemistry, and assessments of body composition. All participants were provided with documents that contained nutritional guidelines, which included recommendations aimed at reducing sugar, salt, and fat consumption. During the study, we maintained contact with participants through weekly phone interviews and the LINE application to monitor compliance, adverse effects, and to record 24‐h dietary recall information. At the conclusion of the study, additional data were collected, including blood biochemistry, dietary intake, and measurements of body composition.

### 2.5 Statistical Analysis

The research data were analyzed using IBM SPSS Statistics for Windows, Version 25.0 (IBM Corp., Armonk, NY, USA). Descriptive statistics were applied to general characteristics, health information, and nutritional assessments. Quantitative data are presented as means and standard deviations (mean ± SD), while categorical variables are expressed as frequencies (*N*), percentages, and 95% confidence intervals. An intention‐to‐treat (ITT) analysis was performed, and between‐group comparisons were adjusted for baseline values using analysis of covariance (ANCOVA). Per‐protocol (PP) results were consistent with the ITT findings. The choice of inferential statistical methods was guided by the study's objectives. To assess mean differences among the three groups (two intervention arms and one control), two‐way ANOVA and repeated‐measures analysis were employed. All statistical tests were two‐tailed, with significance set at *p* < 0.05.

## 3 Results

A total of 65 participants met the inclusion criteria for this study. These participants were subsequently randomized into three groups as part of a randomized controlled trial designed as a double‐blind experiment, resulting in 21 to 22 individuals in each group. The participants were monitored for a period of 60 days. At the conclusion of the study, data from 60 participants (20 from each group) were incorporated into the analysis (refer to Table 1), with an average age of 46 years. At baseline, the average height, weight, body mass index (BMI), and waist‐hip ratio of the participants were recorded as 157.4 cm, 72.4 kg, 29.2 kg/m^2^, and 0.9, respectively. Table 2 provides the mean (±SD) of energy and nutrient intake at baseline prior to administering COS. Mean energy intake, as well as the amounts of carbohydrates, protein, and fat, were documented at 1577 kcal/day, 174.2 g/day, 74.2 g/day, and 65.8 g/day, respectively. Intakes of sugar and sodium surpassed the recommended levels (Jiang et al. 2018), which are 55 g/day for sugar and 3000 mg/day for sodium.

**TABLE 1:** Baseline and demographic characteristics of participants (mean ± SD).

Parameters	Control (*n* = 20)	COS 1.5 g/day (*n* = 20)	COS 3 g/day (*n* = 20)	Total (*N* = 60)
Demographics				
Age (year)	46.0 ± 8.0	46.0 ± 9.0	46.0 ± 8.0	46.0 ± 8.0
Anthropometry				
High (cm)	156.4 ± 4.1	156.9 ± 7.0	158.9 ± 6.7	157.4 ± 6.1
Weight (kg)	69.6 ± 8.8	74.5 ± 15.8	73.2 ± 7.9	72.4 ± 11.4
BMI (kg/m^2^)	28.4 ± 3.2	30.2 ± 5.9	29.1 ± 3.4	29.2 ± 4.3
Waist/hip ratio	0.9 ± 0.0	0.9 ± 0.1	0.9 ± 0.1	0.9 ± 0.1
Biochemistry				
Total cholesterol; TC (mg/dL)	262.5 ± 7.7	259.2 ± 7.7	256.2 ± 7.7	259.3 ± 34.0
LDL‐CHOL; LDL (mg/dL)	172.9 ± 7.6	171.7 ± 7.6	168.0 ± 7.6	170.9 ± 33.3
HDL‐CHOL; HDL (mg/dL)	62.7 ± 3.2	59.6 ± 3.2	62.1 ± 3.2	61.5 ± 14.1
Triglyceride; TG (mg/dL)	134.7 ± 13.0	138.6 ± 13.0	130.6 ± 13.0	134.6 ± 57.4
Fasting blood sugar; FBS (mg/dL)	95.0 ± 4.8	104.7 ± 4.8	108.1 ± 4.8	102.6 ± 21.6
SGOT (mg/dL)	19.9 ± 1.0	22.1 ± 1.0	20.1 ± 1.0	20.7 ± 4.3
SGPT (mg/dL)	19.5 ± 1.7	20.2 ± 1.7	21.0 ± 1.7	20.2 ± 7.3

**TABLE 2:** Mean of energy and nutrients consumption at baseline (mean ± SD).

Parameters	Control (*n* = 20)	COS 1.5 g/day (*n* = 20)	COS 3 g/day (*n* = 20)	Average (*N* = 60)	%Thai RDI^a^
Energy intake (kcal/day)	1546 ± 366	1853 ± 1436	1332 ± 514	1577 ± 262	88.0
Carbohydrate (g/day)	183.2 ± 68.9	194.5 ± 74.3	145.0 ± 61.7	174.2 ± 25.9	77.4
Protein (g/day)	69.2 ± 27.8	91.3 ± 137.2	62.18 ± 26.0	74.2 ± 15.2	96.4
Fat (g/day)	58.6 ± 22.8	78.9 ± 96.1	59.8 ± 37.1	65.8 ± 11.4	101.2
Dietary fiber (g/day)	9.9 ± 6.9	10.1 ± 7.2	7.7 ± 3.7	9.2 ± 1.3	37.0
Sugar (g/day)	61.6 ± 54.5	63.9 ± 51.9	41.9 ± 24.7	55.8 ± 12.1	85.9
Na (mg/day)	2861 ± 2770	3168 ± 4702	3132 ± 2683	3054 ± 168	153.0

^a^ Thai RDI recommend that the amount of total energy intake in adult women aged 19–60 years should be average 1500–1800 kcal/day, carbohydrate 45.0%–65.0% of total energy intake, protein 15.0%, Fat 20.0% of total energy. Dietary fiber intake should be more than 25.0 g/day, sugar intake less than 65.0 g/day, and sodium should be less than 2000 mg/day.

Throughout the 60‐day supplementation phase, participants consumed COS prior to meals three times daily. The study demonstrated an average compliance rate of 96.0% verified via capsule counts, considered acceptable (≥ 90.0%), with no adverse events related to COS reported. The impact of COS on the lipid profile of overweight or obese Thai women was evaluated through serum lipid analysis. Blood biochemistry data for the 60 participants are summarized in Table 3. At the end of the study, there were no significant differences in blood biochemistry parameters among the groups.

**TABLE 3:** Comparison of blood biochemistry between baseline‐ending changes in three study groups (mean ± SD) (*N* = 60).

Variables	Control (*n* = 20)		COS 1.5 g/day (*n* = 20)		COS 3 g/day (*n* = 20)		*p*	*p*
	Baseline	Ending	Baseline	Ending	Baseline	Ending		
								Bootstrap (*N* = 1000)
Total cholesterol; TC (mmol/L)	6.8 ± 1.0	7.0 ± 1.3	6.7 ± 0.7	6.6 ± 0.8	6.6 ± 0.9	6.8 ± 1.0	0.520	0.473
LDL‐CHOL; LDL (mmol/L)	4.5 ± 1.1	4.5 ± 1.3	4.4 ± 0.6	4.1 ± 0.7^a^	4.4 ± 0.8	4.3 ± 1.0	0.387	0.418
HDL‐CHOL; HDL (mmol/L)	1.6 ± 0.4	1.8 ± 0.4^b^	1.5 ± 0.4	1.7 ± 0.4^b^	1.6 ± 0.3	1.7 ± 0.4	0.284	0.185
Triglyceride; TG (mmol/L)	1.5 ± 0.7	1.5 ± 0.7	1.6 ± 0.6	1.8 ± 1.0	1.5 ± 0.6	1.7 ± 0.6	0.284	0.225
Fasting blood sugar; FBS (mmol/L)	5.3 ± 0.4	5.7 ± 0.5	5.8 ± 1.6	6.5 ± 2.6^b^	6.0 ± 1.3	6.5 ± 2.9	0.488	0.598
SGOT (U/L)	19.9 ± 4.0	22.1 ± 6.4	22.1 ± 5.1	21.1 ± 3.8	20.1 ± 3.7	22.9 ± 9.9^b^	0.156	0.043^b^
SGPT (U/L)	19.5 ± 7.3	21.1 ± 9.7	20.2 ± 7.2	19.5 ± 7.5	21.0 ± 7.6	22.4 ± 10.5	0.573	0.489

^a^ *p*‐value for difference within group using *F*‐tests for pairwise comparisons (*p*‐value < 0.010).

^b^ *p*‐value for difference within group using *F*‐tests for pairwise comparisons (*p*‐value < 0.050).

Within‐group comparisons of blood biochemistry parameters revealed significant changes exclusively in the control group, characterized by a decrease in high‐density lipoprotein (HDL) levels (*p* < 0.050) (Table 3). Conversely, in the group receiving 1.5 g/day of COS, low‐density lipoprotein (LDL) levels significantly dropped from baseline (*p* < 0.010), while mean HDL levels demonstrated a statistically significant increase from baseline (*p* < 0.050). These findings are consistent with those reported by Choi et al. (2012), who investigated the effects of COS on the reduction of plasma lipid levels among healthy male participants, both smokers and non‐smokers. Participants in their study were administered COS with a molecular weight (MW) of less than 1000 Da at a dosage of 500 mg in water, taken twice daily before breakfast and dinner over 6 weeks. Results indicated significant reductions in total cholesterol (TC) levels (*p* < 0.010) and LDL levels (*p* < 0.050) from baseline for both groups. However, no significant changes in lipid profiles were observed in the group receiving 3 g/day of COS.

Anthropometric measurements were categorized based on the participants' BMI (Table 4). At baseline, the mean BMI was recorded at 29.2 ± 4.3 kg/m^2^, suggesting that a majority of participants were classified as overweight or obese. After the 60‐day COS supplementation, no significant differences in anthropometric parameters were detected across the groups. These findings are in alignment with those of Choi et al. (2012), who similarly reported unchanged body weight and BMI levels in their study. Additionally, these results corroborate a study conducted by Mhurchu et al. (2004), which examined the effects of chitosan on body weight and found no significant differences between the chitosan and placebo groups.

**TABLE 4:** Comparison of anthropometric measurements within each group from baseline to the end of the study across three study groups (mean ± SD) (*N* = 60).

Variables	Control (*n* = 20)			COS 1.5 g/day (*n* = 20)			COS 3 g/day (*n* = 20)		
	Baseline	Ending	*p*	Baseline	Ending	*p*	Baseline	Ending	*p*
Weight (kg)	69.6 ± 8.8	69.1 ± 9.1	0.230	74.5 ± 15.8	74.4 ± 16.1	0.639	73.2 ± 7.9	73.0 ± 7.4	0.512
BMI (kg/m^2^)	28.4 ± 3.2	28.2 ± 3.3	0.209	30.2 ± 5.9	30.1 ± 5.9	0.500	29.1 ± 3.4	29.0 ± 3.2	0.487
Waist (cm)	92.6 ± 6.3	89.4 ± 10.7	0.037^b^	97.0 ± 10.6	94.7 ± 9.4	0.017^b^	95.3 ± 7.2	91.7 ± 7.2	0.001^a^
Hip (cm)	105.9 ± 5.3	104.5 ± 6.0	0.007^b^	107.9 ± 10.1	107.1 ± 9.7	0.310	107.2 ± 5.7	106.9 ± 6.1	0.713
Waist to hip ratio	0.9 ± 0.0	0.9 ± 0.1	0.057	0.9 ± 0.1	0.9 ± 0.1	0.061	0.9 ± 0.1	0.9 ± 0.1	0.004^b^

^a^ *p*‐value for difference within group using *F*‐tests for pairwise comparisons (*p*‐value < 0.001).

^b^ *p*‐value for difference within group using *F*‐tests for pairwise comparisons (*p*‐value < 0.050).

However, it was noted that at the end of the study, the group consuming 3 g of COS per day experienced a statistically significant reduction in waist circumference from baseline (*p* < 0.001). This observation aligns with a corresponding decrease in waist circumference. Notably, hip circumference did not exhibit significant changes in either intervention group. The control group also demonstrated a significant reduction in waist circumference and hip circumference from baseline (*p* < 0.050). Research by Stubbs and Lee (2004) indicated an association between energy intake and body weight status. Overweight and obese participants reported higher energy intakes compared to their normal‐weight counterparts. This positive correlation between energy intake and body weight status was further substantiated by findings from the WHO MONICA aggregate level analysis, which indicated that increasing energy intake correlates with rising rates of overweight and obesity in European countries (Silventoinen et al. 2004). Similar data from Australia and the United States supports the conclusion that increased energy intake is associated with obesity (Stubbs and Lee 2004).

Furthermore, we observed a notable change in the waist‐to‐hip ratio from baseline to the conclusion of the study across three study groups. Notably, the COS 3 g/day group demonstrated the most significant reduction in the waist‐to‐hip ratio. Data derived from a systematic review and meta‐analysis concerning the effects of chitosan supplementation on body weight and body composition indicated that consumption of chitosan at doses exceeding 2.4 g/day, in shorter‐duration studies lasting less than 12 weeks, in parallel design, and involving participants classified as obese or overweight, yielded positive effects on body composition.

Chitosan is a dietary supplement recognized for its potential to reduce body weight and improve lipid profiles through the binding of gastrointestinal fat (Huang et al. 2020). Moreover, COS supplementation has been associated with reductions in body weight and body fat by inhibiting the activity of pancreatic lipase (Kang et al. 2012; Sutthasupha and Lungkaphin 2020), enhancing thermogenic capacity (Lee et al. 2010), and suppressing lipid accumulation along with adipocyte differentiation (Cho et al. 2008) in animal models. Nevertheless, the mechanistic pathways in human subjects remain inadequately defined.

## 4 Discussion

Previous studies have established that chitosan may effectively lower total cholesterol and LDL cholesterol levels in human subjects (Bokura and Kobayashi 2003; Jaffer and Sampalis 2007). Furthermore, various studies conducted on animal models have illustrated that COS exert positive effects on lipid profiles (Huang et al. 2015; Jiang et al. 2018; Pan et al. 2016, 2018). Liao et al. (2007) undertook a study to assess the hypocholesterolemic effects of water‐soluble chitosan (with a molecular weight ranging from 30 to 50 kDa) versus water‐insoluble chitosan (with a higher molecular weight of 100 to 150 kDa). Their findings revealed that water‐insoluble chitosan was superior in its cholesterol‐lowering effects compared to its water‐soluble counterpart. This disparity in efficacy may be attributed to the greater molecular weight of chitosan, which encompasses numerous cationic residues capable of physically binding to dietary lipids and bile acids. Consistent with these findings, Shing‐Hwa et al. (2021) explored the effects and mechanisms of chitosan and COS on intestinal absorption, concluding that COS decreased lipid absorption by reducing the expression of fabp2 and fatp2 mRNA. Thus, chitosan mitigates lipid absorption through its physical properties, promoting lipid excretion and inhibiting absorption. It is essential to note, however, that the molecular weight of the COS used in this investigation was relatively low, allowing for intestinal absorption (Huang et al. 2015; Pan et al. 2018). The efficacy of low molecular weight COS in reducing lipid absorption warrants further investigation over an extended period, likely exceeding the 60‐day duration of this study.

This study corroborates the findings of Stubbs and Lee (2004), which assessed the effects of COS on the reduction of plasma lipid levels in healthy individuals (both smokers and non‐smokers). Participants were administered COS with a molecular weight of less than 1000 Da for a duration of 6 weeks at a dosage of 500 mg in water, twice daily before meals. The results indicated a significant reduction in total cholesterol (*p* < 0.010) and LDL cholesterol (*p* < 0.050) from baseline measurements for both groups. While Shing‐Hwa et al. (2021) examined the effects and mechanisms of chitosan and COS on hepatic lipogenesis, they reported that plasma SGOT and SGPT activities were elevated in the COS group compared to the control groups (Liu et al. 2021).

It is important to acknowledge that this study did not control for the participants' typical dietary intake or physical activity levels. The observed differences in baseline caloric intake were not statistically significant and likely reflect natural variation in free‐living dietary habits. All participants were instructed to maintain their usual dietary routines throughout the study. This presents a limitation, as the timing of the study coincided with a lengthy holiday period, potentially leading to increased food consumption. The heightened total energy intake may correlate with body weight status, as indicated by Stubbs and Lee (2004), who noted that energy intake is linked to body weight. Participants classified as overweight or obese tended to have higher energy consumption than those of normal weight. The WHO MONICA aggregate level analysis supports this correlation, revealing a positive relationship between energy intake and body weight status, particularly in European countries (Silventoinen et al. 2004). Data from Australia and the United States further demonstrate a similar association between increased energy intake and obesity (Mhurchu et al. 2004). According to the Thai Recommended Daily Intakes (Thai RDI) (Ministry of Public Health 2020), the total energy intake for women aged 19 to 60 years should range from 1500 to 1800 kcal/day, with carbohydrates accounting for 45.0% to 65.0% of total energy intake, protein constituting 15.0%, and fat comprising 20.0%. Prior to the study, participants in the control group reported calorie intake below the recommended Thai RDI levels. Therefore, the application of COS for blood lipid management and body composition should be considered in conjunction with dietary patterns and physical activity.

## 5 Conclusions

This study evaluated the effects of chitosan supplementation (COS) on overweight or obese Thai women. Participants ingested COS 15 to 30 min prior to meals, three times daily. A repeated measures design was employed over a 60‐day period, involving 65 Thai women classified as overweight or obese, with a body mass index (BMI) of 23.0 kg/m^2^ or greater, waist circumference of 80.0 cm or more, and aged between 30 and 59 years. Inclusion criteria also required a total cholesterol level exceeding 5.2 mmol/L or a low‐density lipoprotein cholesterol (LDL‐C) level above 3.4 mmol/L. The participants were randomly assigned to three groups, receiving either COS at dosages of 1.5 or 3 g/day, or a placebo. Throughout the study, participants were instructed to maintain their regular dietary habits.

COS supplementation was demonstrated to be safe and exhibited potential lipid‐modulating and body composition effects, particularly at the 1.5 g/day dosage. In this group, within‐group analyses revealed a statistically significant reduction in LDL‐C and an increase in HDL. Additionally, a reduction in waist circumference was observed in the 3 g/day group; however, these changes did not reach statistical significance in between‐group comparisons. No significant effects were observed on serum glutamic‐oxaloacetic transaminase (SGOT) levels. As the study was conducted under free‐living conditions without strict dietary or physical activity controls, the findings should be interpreted with caution. Further research involving larger sample sizes, extended intervention periods, and controlled lifestyle variables is warranted to confirm the clinical relevance of COS supplementation.

## Author Contributions

**Natnaree Mayang:** data curation (equal), formal analysis (supporting), funding acquisition (equal), project administration (lead), writing – original draft (equal). **Kitti Sranacharoenpong:** conceptualization (lead), data curation (lead), formal analysis (supporting), funding acquisition (equal), investigation (lead), methodology (lead), project administration (equal), supervision (lead), writing – original draft (lead), writing – review and editing (lead). **Siriyupa Netramai:** investigation (equal), methodology (equal), writing – review and editing (equal). **Korapat Mayurasakorn:** conceptualization (equal), investigation (equal), writing – review and editing (equal). **Magnus Bergkvist:** writing – review and editing (equal).

## Funding

This research was funded by NSRF via the Program Management Unit for Human Resources & Institutional Development, Research and Innovation (grant number MOU‐CO‐2564‐13555‐TH).

## Ethics Statement

The study was approved by the committee of Mahidol University central‐IRB, Mahidol University, and the committee of the Human Research Protection Unit, Faculty of Medicine, Siriraj Hospital, Mahidol University, Thailand (MU‐MOU 2021/457.2610).

## Consent

Written informed consent was obtained from all participants.

## Conflicts of Interest

The authors declare no conflicts of interest.

## Data Availability

The datasets generated and/or analyzed during the current study are not publicly available due to concerns regarding privacy, but select data are available from the corresponding author upon reasonable request.
